# Examination of short and long term complications of thermocautery, plastic clamping, and surgical circumcision techniques

**DOI:** 10.12669/pjms.336.13640

**Published:** 2017

**Authors:** Ahmet Ali Tuncer, Elif Emel Ayar Erten

**Affiliations:** 1 Dr. Ahmet Ali Tuncer, Assistant Professor, Department of Pediatric Surgery, a.Medical Faculty, Afyon Kocatepe University, Afyonkarahisar, Turkey; 2 Dr. Elif Emel Ayar Erten, MD, Clinic of Pediatric Surgery, Hakkari State Hospital, Hakkari, Turkey

**Keywords:** Circumcision, Clamp, Complication, Pediatric, Thermocautery

## Abstract

**Objective:**

In this study, thermocautery, plastic clamping, and conventional (open surgical) circumcision techniques were compared in terms of their complications.

**Methods:**

Male patients who underwent circumcisions between May 2014 and May 2015 in two separate pediatric surgery clinics were retrospectively analyzed using the hospital registry system. These patients were evaluated in terms of age, accompanying pathologies, anesthesia techniques, complication rates, duration of surgery, and circumcision techniques. A statistical analysis of the data was performed, with a P<0.05 considered to be statistically significant.

**Results:**

The patients were divided into three groups according to the circumcision method: conventional surgical circumcision (n=833), thermocautery (n=1011), and plastic clamp (n=218). Complications were observed in 21 cases (1%): bleeding (11), infection (2), trapped penis (6), meatitis (1), and scrotal injury (1). There were significantly fewer complications in the thermocautery technique when compared to the clamping and surgical circumcision techniques. The plastic clamping and thermocautery techniques were superior to a surgical circumcision in terms of the operation time.

**Conclusion:**

The thermocautery circumcision technique can be used easily in both the operating theatre and in designated circumcision rooms, with a lower complication rate, when compared to plastic clamping and surgical circumcisions.

## INTRODUCTION

Male circumcision dates back to ancient Egypt, and is the most common surgical procedure in the world. Circumcisions are done in order to protect individuals against infectious and sexually transmitted diseases, such as HIV, as well as for cultural and religious reasons.^1^ For centuries; people have performed this surgical procedure using various techniques according to the level of socioeconomic development. The surgical techniques used today include the PlastiBell, Gomco clamp, Mogen clamp, Smart clamp, Tara clamp, Shang Ring, and thermocautery.^2^,^3^ The diversity of circumcision techniques is the result of the search for more practical, cheaper, safer, and less complicated methods. Thermocautery technique is a recent technique and is being used for the last ten years. It doesn’t have a wide spread use because many surgeons consider its effects similar to the effects of monopolar cautery. It was shown that thermocautery technique leads to less tissue damage compared to electrocautery techniques in our previous paper.^4^

In this study, the complications of circumcisions performed by pediatric surgeon specialists using three different techniques in two different hospitals in Turkey were investigated retrospectively.

## METHODS

This research was carried out in accordance with the Helsinki declaration rules, with the approval of the local ethics committee. All procedures performed in studies involving human participants were in accordance with the ethical standards of the institutional and/or national research committee and with the 1964 Helsinki declaration and its later amendments or comparable ethical standards. Written informed consents were obtained from patient’s parent for accompanying images.

### Study design and circumcision techniques

Those male patients who applied to the Hakkari State Hospital and Yuksekova State Hospital pediatric surgery clinics between May 2014 and May 2015 for circumcisions were analyzed retrospectively using the hospital registry system. The patients were evaluated in terms of age (0–12 months, 1–5 years, 6–11 years, and 12–18 years), accompanying pathologies, anesthesia techniques, surgery duration, complication rates, and circumcision techniques.

Each circumcision was performed by a pediatric surgeon and an assistant health professional under sterile conditions in the operating room or circumcision room (a semi-sterilized room in the clinic where small surgical interventions are conducted). Those patients with additional inguinal region, pathologies underwent surgery for both the circumcision and inguinal pathology under general anesthesia in the operating room. For those patients circumcised in the circumcision room, a penile block and local infiltration anesthesia (penile ring block, penile dorsal nerve block) were used with Bupivacaine HCl (Marcaine 0.5%; AstraZeneca, Istanbul, Turkey) and prilocaine HCl (Citanest 2%; AstraZeneca, Istanbul, Turkey). Each circumcision was performed using a classical surgical circumcision technique, thermocautery, or aplastic clamp (Alisklamp).

For the classical surgical circumcision (conventional), the foreskin was pulled up with a clamp and the outer skin and secondly mucosa was cut using scissors. After controlling the bleeding, the skin and mucosa were sutured using 5/0 absorbable suture material. A medical dressing was applied.

An Alisklamp (Abagrup Health Services Ltd., Ankara, Turkey) was used for the plastic clamp technique, and the clamp size was chosen according to the diameter of the patient’s penis. The device was placed onto the glans, and the preputium was pulled up over the device. After adjusting the mucosal and skin lengths to be cut, the clamp was tightened over the device. The skin and mucosa were then excised from the distal part of the clamp with a scalpel. The clamp was removed on the 4^th^ day after the surgery.

**Fig. 1:**
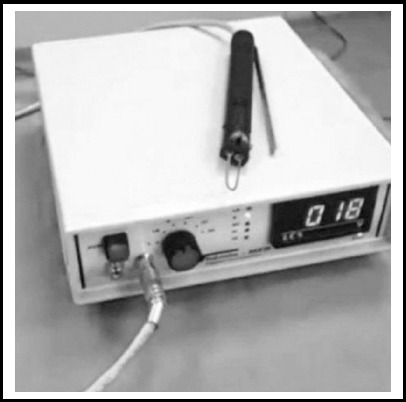
Thermocautery device.

**Fig.2:**
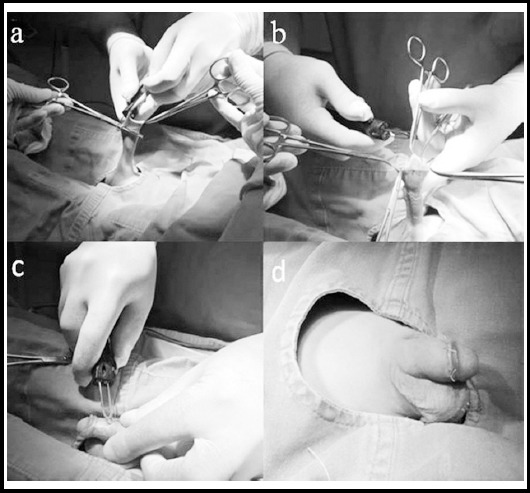
Circumcision with thermocautery technique. a, b)The foreskin was pulled up with the clamps, and the outer skin and mucosa were cut using a thermocautery device. c) Cauterization of dorsal vein; no bleeding was seen in the incision line after cutting the skin and mucosa. d) Postoperative appearance of the penis.

For the thermocautery method, a digital thermocautery device (Thermo-Med TM 802-B; Thermo Medikal, Adana, Turkey) with 6 different temperature settings was used (Fig.1). This circumcision was performed the same way as the surgical circumcision; however, the cutting and cauterization were done using a thermocautery device. The cutting was performed via the appropriate heat adjustment according to the age of the child and the thickness of the preputium. Hemorrhage control was performed with the thermocautery device, and the skin-mucosa integrity was ensured using 5/0 absorbable suture material (Fig.2).

Surgeon 1 used thermocautery in all of the circumcisions he performed, while surgeon 2 used either the surgical circumcision or Alisklamp technique. The choice of technique depended entirely on the experience of the individual surgeon. Following each circumcision, routine warm sitz baths and daily medical dressings were recommended. In addition, epithelizing cream was recommended for those patients with phimosis.

Any trauma to the penis, urethra, or scrotum was considered to be a perioperative complication. Bleeding necessitating suture ligation or cauterization and infections were considered to be early complications. Late complications included meatitis and a buried penis. Although all of the patients were recruited for routine control on the 10^th^ postoperative day, they were also warned about the late complications of a circumcision, and asked if any outpatient admissions occurred.

### Statistical analysis

The data obtained was transferred to a computer and assessed with the help of the Statistical Package for Social Sciences (SPSS) version 19.0 (SPSS Inc., Chicago, IL, USA). The chi-squared test was used to evaluate the categorical data and the Mann-Whitney U test was used to evaluate the quantitative variables. The distribution of the data was tested by using one of the normality tests, such as the Shapiro-Wilk’s test, in the comparison of the surgical time. The Kruskal-Wallis test was used for the nonparametric group comparisons when the data was not normally distributed. The Dunn test was used as a post-hoc test when different groups were determined. A P<0.05 was considered to be statistically significant.

## RESULTS

In total, 2,062 male patients were circumcised between May 2014 and May 2015 in the Hakkari State Hospital (n=1,051) and Yuksekova State Hospital (n=1,011) pediatric surgery clinics. Inguinal pathologies were observed in 132 patients [inguinal hernia (n=96, 4.6%) and undescended testis (n=36, 1.7%)], while 1,930 (93.6%) patients did not have any additional pathologies. The mean age range (mean ± SD) of the patients included in this study was2.95±3.1 years old (range 0–18). In total, 1,712 (83%) of the patients were circumcised when they were under 5 years of age. The age distribution of the patients was as follows: 0–12 months (n=523), 1–5 years (n=1,189), 6–11 years (n=302), and 12–18 years (n=48).

The patients were divided into three groups according to the circumcision technique: classical surgical technique (n=833, 40.4%), thermocautery technique (n=1,011, 49.0%), and clamp technique (n=218, 10.6%). Overall, 1,919 patients (93.1%) were operated on under local anesthesia and 143 patients (6.9%) were operated on under general anesthesia. The surgical processing times of the groups were as follows: classical surgical technique 14.38±2.91 minutes, thermocautery technique 5.02±1.32 minutes, and Alisklamp 4.05±1.0 minutes. All three techniques were statistically different from each other in terms of the duration of surgery (P=0.000 for all groups).

**Fig.3:**
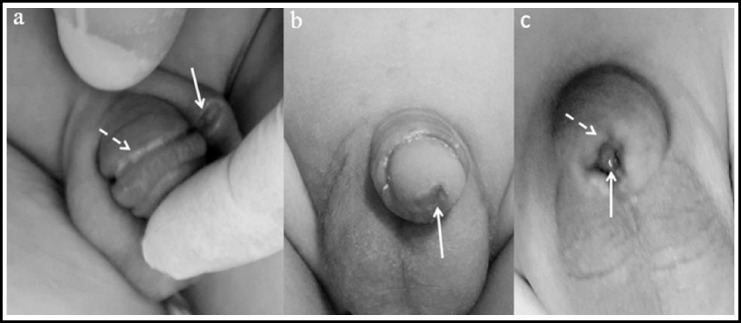
Complications. a) Alisklamp complication showing scrotal injury due to extensive cutting of the preputium with a scalpel after clamping. Arrow: circumcision line after the clamp removal. Dashed arrow: scrotal injury. b) Arrow: meatitis developed three months after the thermocautery application. c) Trapped penis after the open surgery technique. The same complication also developed after the Alisklamp and thermocautery techniques. Arrow: trapped glans. Dashed arrow: narrowing preputium in front of the glans penis.

When the patients were evaluated for long and short term complications, they were observed in 21 cases (1%). Of these, 11 had hemorrhages (0.5%), two had infections (0.1%), six had buried/trapped penises (0.3%), one had meatitis (0.01%), and one had a scrotal injury (0.01%) (Fig.3). The complication types according to the circumcision techniques are shown in Table-I. Complications were observed in 10 (1.2%) of the 833 patients that underwent circumcisions with the classical surgical technique, with hemorrhage being the most common complication (n=8, 0.96%). The thermocautery technique was used in 1,011 patients, with 4 (0.4%) complications observed. In addition, complications were observed in 7 of the 218 circumcisions performed with the clamp technique (3.2%). A buried penis/excessive foreskin (n=4, 1.83%) was the most common complication. Overall, thermocautery was superior to the clamp and open surgical techniques in terms of the complications (P =0.001 and P =0.048, respectively).

**Table-I:** The complication rates according to the circumcision techniques.

*Complications*		*Techniques*		

		*Thermocautery*	*Clamp*	*Classical surgery*
Perioperative Early postoperative	Scrotal injury (n=1) Bleeding (n=11)	1	1 2	8
	Infection (n=2)	1		1
Late postoperative	Buried penis/Secondary phimosis (n=6)	1	4	1
	Meatitis (n=1)	1		
	P for chi-squared test		P_T_-P_C_:0.001	P_T_-P_S_:0.048

P_T_: P for thermocautery, P_C_: P for clamp, P_S_: P for classical surgery.

There was a statistically significant difference between the frequency of complications and the anesthesia technique (P=0.011). Moreover, all of the complications occurred in those circumcisions performed under local anesthesia. However, there was no difference regarding the complication rate when the age groups were compared in terms of the type of the surgical technique (P >0.05).

## DISCUSSION

Traditionally, Turkish parents generally prefer to have male circumcisions performed at 6–7 years of age.^5^ However, due to the beliefs that healing is better in infants and that infants feel less pain, recent studies have shown that circumcisions have begun to shift to younger ages (as in our work).^6^

When all of the techniques used in our study were examined, a 1.01% complication rate was observed. Previous literature has reported a wide range of complications, ranging from 0.1% to 35%.^7^ Our low complication rate could be explained by the fact that the surgeons were experienced in the applied techniques.

Saracoglu et al. reported that circumcisions done with the thermocautery technique led to less bleeding, with a shorter operation time, when compared to the conventional method.^8^ When compared to the other techniques, a surgical circumcision causes more bleeding from the foreskin and mucosa. Since additional effort and a separate device are required for coagulation, it is difficult to apply it outside the operating room. However, thermocautery and the clamp can be applied in sterile conditions in a circumcision room. In both techniques, there is less hemorrhaging during the circumcision. Because the preputium distal to the clamp is cut, no bleeding occurs unless the clamp is removed or broken, but there is a risk of hemorrhage on the 4^th^ day when the clamp is removed. The thermocautery device also provides coagulation during the circumcision process, because it converts electric energy into heat energy.^2^,^9^ Minimal interventions is only needed in dorsal vein or frenular artery bleeding during the circumcision. When the thermocautery and clamping techniques are used, time is not lost with coagulation; therefore, these techniques are more advantageous when compared to surgical circumcision in terms of the duration. However, secondary phimosis due to excessive burning of the skin may occur when the device is used at high temperatures by inexperienced persons. In our study, only one thermocautery-derived secondary phimosis was observed.

In electrocautery circumcisions, the electrical current passes directly through the penile tissue, and if the preputium is cut by electrocautery after being clamped, there can be total phallic loss (grade V injuries) due to direct contact between the cautery and the clamp.^10^,^11^ Electrocautery and thermocautery work on different principles. For example, the thermocautery device supplies the electrical energy by turning it into heat energy instead of passing it through the tissue. However, the electric current in electrocautery passes through the tissue and is blamed for various complications. Tuncer et al. (principle author of present article) found that the thermocautery technique was better in terms of wound healing and the depth of the injury when compared to the bipolar and electrocautery techniques in an experimental circumcision model in rats.^4^

In the clamp technique, the patient carries the apparatus on his penis for 4 days. More edema occurs in the penile skin proximal to the clamp when compared to the surgery and thermocautery techniques. When the clamp is removed from the penis on the 4^th^ day, he may experience a second fear of circumcision during this procedure. There is no second trauma in the surgical and thermocautery techniques because of the use of absorbable sutures. With regard to the healing time, surgical and thermocautery circumcisions generally show complete healing within 3–10 days, while it takes 10 days in the case of clamps. In a study conducted by Şenel et al., wound healing was observed at an average of 25 days with the clamp technique in adults.^12^ The Alisklamp is disposable and offers superior hygiene and less infection when compared to the other techniques, as seen in our series; however, it can be disadvantageous in terms of its cost.

There was a complication rate of 2.47% in the mass circumcisions in which Şenel et al. used plastic clamps (novel plastic clamps). The most common complications were buried penises and excessive foreskins.^13^ In our study, a 3.21% complication rate was observed in the clamp technique. The most common complications were buried penises and excessive foreskins (1.83%).

Surgical periods varying from 3.6 to 11 minutes have been reported in the literature when using the clamp technique.^13^,^14^ In the open surgical technique studies, it was reported that the surgical process took longer than 20 minutes.^15^ In accordance with the literature, in our study, the clamp and thermocautery techniques were found to be superior to the classical surgical technique.

In terms of cost, one Alisklamp for each clamp circumcision, the thermocautery device and one absorbable suture for each thermocautery technique, and one absorbable suture for each open surgery were the considered costs when the common expenses were ignored (personnel, sterility, surgical set, and medical dressing). According to the normal depreciation account of the thermocautery device based on the data of the Ministry of Finance of the Republic of Turkey, the cost per circumcision was 0.116 USD (device price/amortization year/annual circumcision: 708/6/1,011=0.116 USD).^16^ However, the disposable clamp cost was 6.25 USD. These calculations were made using the January 2015 exchange rate of 1 USD=2.40 TL.^17^

There are not many studies in the literature about the thermocautery technique in circumcisions. Moreover, few studies have short term results, and long term follow up results are not available.^18^ In our study, a buried penis and secondary phimosis were the long-term complications due to the surgical circumcision, thermocautery and clamp techniques, and surgical treatments were performed. In addition, a meatitis was observed in the postoperative third month after one thermocautery technique. This complication was medically treated.

### Limitations

Surgical techniques are best compared in a prospective randomized fashion but this is a retrospective study. Also thermocautery technique was performed by the first author and the other techniques were performed by the second author. It may have an impact on differences seen in complication rates. Both authors have a similar experience and education during their training.

## CONCLUSION

If the thermocautery technique is used by experienced personnel maintaining sterile conditions in operating or circumcision rooms, it is a good choice in terms of the time, cost, and complication rate.
